# Artificial intelligence guided Raman spectroscopy in biomedicine: Applications and prospects

**DOI:** 10.1016/j.jpha.2025.101271

**Published:** 2025-03-19

**Authors:** Yuan Liu, Sitong Chen, Xiaomin Xiong, Zhenguo Wen, Long Zhao, Bo Xu, Qianjin Guo, Jianye Xia, Jianfeng Pei

**Affiliations:** a Department of Pharmaceutical Engineering, Beijing Institute of Petrochemical Technology, Beijing, 102627, China; b Beijing Key Laboratory of Enze Biomass and Fine Chemicals, Beijing Institute of Petrochemical Technology, Beijing, 102627, China; c Chongqing Key Laboratory of Intelligent Oncology for Breast Cancer, Chongqing University Cancer Hospital and School of Medicine, Chongqing University, Chongqing, 400000, China; d Academy of Artificial Intelligence, Beijing Institute of Petrochemical Technology, Beijing, 102627, China; e Tianjin Institute of Industrial Biotechnology, Chinese Academy of Sciences, Tianjin, 300074, China; f Center for Quantitative Biology, Academy for Advanced Interdisciplinary Studies, Peking University, Beijing, 100871, China

**Keywords:** Artificial intelligence, Deep learning, Raman spectrum, Process analytical technology, Bioprocess, Biomedicine

## Abstract

Due to its high sensitivity and non-destructive nature, Raman spectroscopy has become an essential analytical tool in biopharmaceutical analysis and drug development. Despite of the computational demands, data requirements, or ethical considerations, artificial intelligence (AI) and particularly deep learning algorithms has further advanced Raman spectroscopy by enhancing data processing, feature extraction, and model optimization, which not only improves the accuracy and efficiency of Raman spectroscopy detection, but also greatly expands its range of application. AI-guided Raman spectroscopy has numerous applications in biomedicine, including characterizing drug structures, analyzing drug forms, controlling drug quality, identifying components, and studying drug-biomolecule interactions. AI-guided Raman spectroscopy has also revolutionized biomedical research and clinical diagnostics, particularly in disease early diagnosis and treatment optimization. Therefore, AI methods are crucial to advancing Raman spectroscopy in biopharmaceutical research and clinical diagnostics, offering new perspectives and tools for disease treatment and pharmaceutical process control. In summary, integrating AI and Raman spectroscopy in biomedicine has significantly improved analytical capabilities, offering innovative approaches for research and clinical applications.

## Highlights

• Raman's non-destructive, high-sensitivity drives biomedical/biopharmaceutical applications.
• PLS/SVM remain key chemometric tools; ML/DL enhance analysis complexity & efficiency.
• DL models (CNN, LSTM, GAN) improve pattern recognition & predictive accuracy.
• Transformers enable big data processing/feature extraction, rising in Raman spectroscopy.

## Graphical abstract

**Figure:**
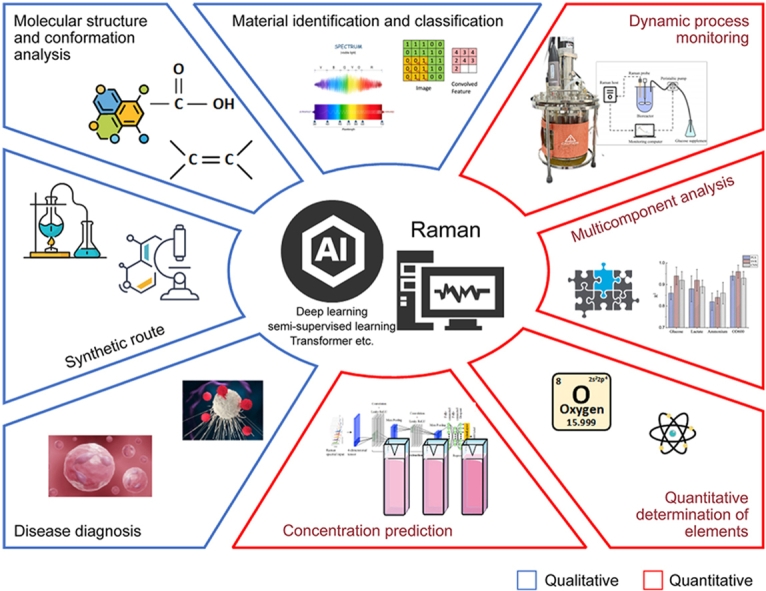

## 1 Introduction

In 1928, Indian physicist C.V. Raman and his students [1,2] discovered the phenomenon of Raman scattering and revealed that when monochromatic light is directed at a substance, some photons are absorbed by the molecules and cause molecular vibrations, while the scattered photons carry information about the molecular structure. This discovery laid the foundation for the evolution of Raman spectroscopy technology. Specially, Raman scattering phenomenon has driven the development of Raman spectroscopy, which provides a powerful tool for the early diagnosis and treatment of diseases in biomedical field through rapid and non-destructive testing methods. Raman spectroscopy, in contrast to the molecular functional group analysis provided by Fourier transform infrared spectroscopy (FTIR) [3], has evolved with technological advancements such as enhanced sensitivity and resolution from laser light sources, enabling its extensive use in the biopharmaceutical field for applications like quality control in drug manufacturing [4]. For example, in the identification of drug polymorphs, Raman spectroscopy can effectively distinguish different polymorphs by detecting the subtle changes in the vibrational modes of drug molecules, which is crucial for ensuring the stability, bioavailability, and efficacy of drugs.

On the one hand, Raman spectroscopy has shown extensive potential applications in biopharmaceutical fields due to its non-destructive and high-sensitivity analytical nature [5], for example, Raman spectroscopy can be used for component distribution analysis. For example, in the distribution uniformity study of active ingredients in freeze-dried preparations, Raman spectroscopy can present the distribution imaging of each component in the preparation through chemical microscopic imaging technology, which is helpful for evaluating product quality and optimizing processes. On the other hand, Raman technology can characterize drug structures, analyze drug forms, control drug quality, and study drug interactions, effectively supporting the drug synthesis and quality control. For example, Raman spectroscopy offers critical molecular structural information, optimizing the drug synthesis pathways [6]. For drug structural characterization, vibrational spectroscopy analysis accurately determines the chemical bond types, positions, and conformations in drug molecules [7]. Recently, Raman spectroscopy can also be applied to drug form analysis, revealing the drugs' crystal structures and morphological characteristics and optimizing their physicochemical properties [8], for example, in the synthesis of aspirin, Raman spectroscopy is used to track the formation of intermediates and products during the reaction process, thereby optimizing reaction conditions and improving yield.

In quality control, Raman spectroscopy, as a rapid and efficient analytical technique, monitors the composition content, detects impurities, and ensures batch-to-batch consistency of drug products. Furthermore, Raman spectroscopy can be employed in drug interaction studies to elucidate the mechanisms of interaction between drugs and biomolecules, aiding in a deeper understanding of the drugs' pharmacological and toxicological properties. However, Raman spectroscopy data usually contains a large amount of noise and complex background information, which poses a great challenge to traditional analysis methods. Artificial intelligence technology (such as regression models or classification algorithms) has strong anti-interference ability and feature extraction ability, and can effectively process this complex data and improve the accuracy of the analysis [9]. Integrating machine learning and deep learning algorithms with Raman spectral data analysis can effectively reduce the complexity of data analysis and provide more comprehensive and efficient methods [10]. Indeed, machine learning algorithms can be applied for the Raman spectral data to reduce data complexity and noise interference, thereby enhancing data quality and analytical efficiency. Through classification algorithms, sample classification and structure identification can be achieved to analyze Raman spectral data qualitatively. In contrast, regression analysis can be used to develop accurate predictive models for quantitative information [11]. Furthermore, deep learning algorithms can identify hidden patterns and regularities in complex Raman spectral data to improve pattern recognition and interpretation [12]. Machine learning-based approaches are expected to harness the advantages of Raman spectroscopy technology, enhance the scientific nature and reliability of data analysis, and provide powerful analytical tools and methods for scientific research and engineering applications.

Partial least squares (PLS) regression is a typical chemometric algorithm widely applied in Raman spectroscopy data analysis [13]. Before model development, spectral data require preprocessing steps such as smoothing filtering, baseline correction, scattering correction, and normalization. PLS is then employed to minimize the prediction errors of response variables, thereby reducing the dimensionality of the dataset, extracting the primary information and features, and simplifying the data complexity to establish predictive models. For instance, He et al. [14] developed a label-free method to measure the extracellular protein kinase (PKA) activity quantitatively. They identified two Raman peaks at 725 and 1,395 cm^−1^ and quantitatively measured PKA activity. Besides, support vector machines (SVM) can classify and identify biological sample features qualitatively. For instance, Dong et al. [15] utilized an SVM classification algorithm in conjunction with portable Raman spectroscopy to develop a rapid detection method for methylenedioxymethamphetamine (MDMA) in human urine. In conclusion, when dealing with large-scale data, PLS and SVM models may face computational resource and time constraints. Especially when the amount of data is huge, the training and prediction speed of the model may become very slow, and even cannot complete the calculation in a reasonable time.

Compared to traditional machine learning, deep learning offers advantages such as automatic feature extraction, handling large-scale data, more robust generalization and adaptability, and the ability to process time-series data [16]. Convolutional neural networks (CNNs) can identify and classify drug components, analyze Raman spectral data feature peaks and spectrum patterns, monitor drug component content for quality control, and identify foreign substances and contaminants to ensure compliance [17]. Based on CNN models, the characteristics of drug formulations in Raman spectral data can be analyzed, and the corresponding formulations can be optimized to enhance stability and efficacy. In continuous production, Raman spectroscopy possesses temporal sequences, and long short-term memory (LSTM) networks, a type of recurrent neural network, can handle long-term dependency relationships in time-series data [18]. In biopharmaceutical processes, LSTM can analyze Raman spectral data series features, predict changes in critical reaction parameters for real-time monitoring and control, and even predict future quality parameter changes based on past spectral data, assisting in production process adjustments and quality control [19]. By learning the patterns of spectral data sequences, future reaction quality parameters can be predicted, guiding production optimization and quality control. Besides, generative adversarial networks (GANs) have also been applied to Raman spectroscopy analysis for data augmentation, feature extraction, and model optimization. GANs comprise generators and discriminators, where the generators produce synthetic data, and the discriminators judge authenticity [20]. In Raman spectroscopy analysis, GANs generate new spectral data to enhance the diversity and scale of the original data set [21], thereby improving model generalization and robustness. By training the generators to learn the distributional features of spectral data, key features can be automatically extracted, reducing the workload of handcrafting feature extractors and enhancing predictive performance. Additionally, GANs have been used for model optimization by training the discriminators to distinguish real and synthetic Raman spectral data. This is achieved by optimizing the model structure and parameters to improve accuracy and efficiency. Moreover, applying the graph neural networks (GNN) and Transformer model in Raman spectroscopy analysis primarily focuses on graphic feature extraction, big data processing and model optimization.

In conclusion, deep learning plays a pivotal role in enhancing the accuracy and efficiency of Raman spectroscopy, particularly in spectral preprocessing, classification, and regression tasks [22]. It also enables advanced applications such as real-time pathogen identification by integrating open-set deep learning with single-cell Raman spectroscopy [23].

## 2 Application of Raman spectroscopy in biomedicine field

**Fig. 1:**
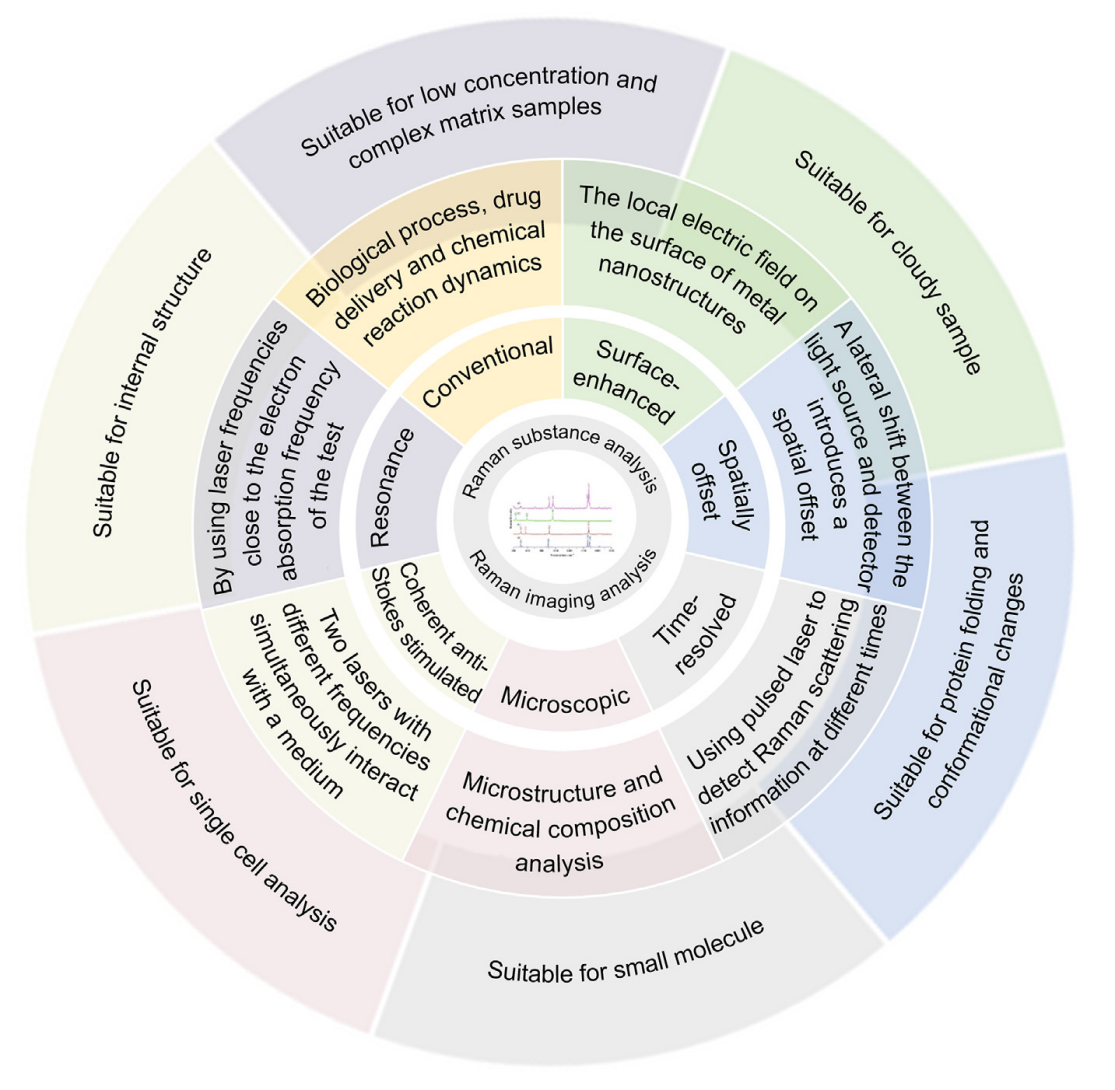
Application of Raman spectroscopy in biomedicine field. A schematic diagram of the application of Raman spectroscopy in the field of biomedicine. A typical Raman spectrum is placed in the center of the image; The second layer is the two most important aspects of Raman spectroscopy technology: substance analysis and imaging analysis; The third layer represented typical Raman spectroscopy techniques; The fourth layer is the mechanism of different Raman spectroscopy techniques; The outermost layer is the applicable fields of them.

Raman spectroscopy and imaging technologies have established themselves as powerful analytical tools in biomedicine, enabling non-invasive, label-free investigation of molecular composition and structural organization [24]. Raman spectroscopy provides detailed vibrational signatures that facilitate real-time cellular analysis, disease diagnostics through biomarker detection, and the study of drug-target interactions. Raman imaging, which combines the spatial resolution of optical microscopy with the chemical specificity of Raman spectroscopy, allows for the generation of high-resolution molecular maps, revealing the spatial distribution of biomolecules such as lipids, proteins, and nucleic acids within tissues [25]. This capability has been demonstrated in applications such as precise delineation of tumor margins and characterization of tissue composition (Fig. 1). Compared to fluorescence imaging, Raman techniques offer intrinsic molecular specificity without the need for exogenous labels, thereby preserving sample integrity and reducing phototoxicity [26]. These advantages make Raman-based methods particularly valuable for studying unmodified biological systems and advancing both fundamental research and clinical applications.

### 2.1 Classification of Raman spectra

Raman spectroscopy is a spectral analysis method based on the Raman scattering principle, which obtains molecular vibration information in samples by detecting the frequency shift of scattered light. In biomedicine, Raman spectroscopy holds significant application value. Among them, the *in situ* Raman spectroscopy technique allows for compositional analysis without damaging the sample, the surface-enhanced Raman spectroscopy technique can enhance signal intensity, and the micro-Raman spectroscopy technique combined with a microscope enables compositional analysis of micro-area samples. Furthermore, the time-resolved Raman spectroscopy technique can dynamically observe chemical reaction processes. In contrast, Raman spectroscopy and imaging technology provide high-resolution compositional distribution images of biological sample surfaces. Each Raman spectroscopy technique has its specific advantages and limitations and suits different types of samples and analysis requirements. Table 1 reports the detailed characteristics of each method [[27], [28], [29], [30], [31], [32], [33], [34], [35], [36], [37], [38], [39], [40], [41], [42], [43], [44], [45], [46], [47], [48], [49], [50], [51], [52], [53]].

**Table 1:** Classification and characteristics of Raman spectroscopy.

Spectral type	Typical applications	Principles	Refs.
Conventional Raman spectroscopy (CRS)	Proteins, nucleic acids, sugars, lipids, cell membranes, organelles, etc.	Raman scattering is generated using excitation light interacting with sample molecules.	[[27], [28], [29], [30]]
Microscopic Raman spectroscopy (MRS)	Cell membrane, small molecule drugs, biomarkers, bacteria, fungi, blood, etc.	An organic combination of optical microscope and Raman spectrometer, which links spectra with spatial information and performs chemical analysis on micrometer-sized objects. Confocal Raman microscopy has good resolution in the 3D space and can measure substances inside containers or characterize samples in 3D.	[[31], [32], [33]]
Surface-enhanced Raman spectroscopy (SERS)	Biomacromolecules, drug molecules, biomarkers, cells and cell components, etc.	The local electric field on the surface of metal nanostructures enhances the Raman scattering signal. The signal is more evident than in conventional Raman spectroscopy.	[[34], [35], [36], [37]]
Spatially Offset Raman spectroscopy (SORS)	Analyze heterogeneous or cloudy samples, such as drug tablets, biological tissues, etc.	A lateral shift between the light source and detector introduces a spatial offset, capturing Raman signals from deeper within the sample. The Raman spectrum closer to the real composition of the sample is obtained by avoiding the interference of the sample surface through spatial migration.	[[38], [39], [40], [41]]
Resonance Raman spectroscopy (RRS)	Detailed structure of proteins, nucleic acids, lipids and sugars, internal chemical and morphological information of cells and tissues, etc.	By using laser frequencies close to or equal to the electron absorption frequency of the test substance, vibrations related to chromophores in the test substance are selectively enhanced. This strategy provides highly selective excitation, further improving the signal-to-noise ratio. This is suitable for complex system analysis.	[[42], [43], [44]]
Time-resolved Raman spectroscopy (TRRS)	Protein folding and conformational changes, drug-protein binding, cell membrane dynamics, fluorescent dyes and biological probes, etc.	Measuring the evolution of Raman spectra over time. Study the dynamic process to obtain the reaction mechanism and kinetic information suitable for complex reaction systems.	[[45], [46], [47]]
Tip-enhanced Raman spectroscopy (TERS)	The structural and conformational changes of proteins, DNA, RNA and other biomolecules were analyzed	Raman signals are significantly enhanced with nanoscale metal tips, enabling high spatial resolution and high sensitivity surface analysis.	[48,49]
Coherent anti-Stokes Raman spectroscopy (CARS) or stimulated Raman spectroscopy (SRS)	Single-cell analysis, chemical reaction kinetics, surface catalytic processes, biomolecule structures, as well as drug development, etc	Two lasers with different frequencies (pump ω1 and Stokes ω2) simultaneously interact with a medium, inducing molecular transitions when their frequency difference matches a Raman vibrational mode (Δω = ω1 - ω2). This process generates phase-matched Stokes (ω3) and anti-Stokes (ω4) photons, with Stokes intensity surpassing that of conventional Raman scattering^a^.	[[50], [51], [52], [53]]

a In SRS, the actual Raman spectral peak positions may vary due to the specific composition of the sample, the state of the sample, and the measurement conditions, such as laser wavelength, sample processing method, etc. ω1: pump frequency; ω2: Stokes frequency; ω3: generated Stokes frequency; ω4: generated Anti-Stokes frequency.

### 2.2 Applications of Raman analysis technology in biomedicine

Raman spectroscopy has been widely utilized in the biomedical field, particularly in drug development, pathological diagnosis, biological specimen analysis, and biomedical imaging. For instance, Raman spectroscopy has been employed to study molecular structures and identify components in pharmaceuticals, offering significant support for drug development. These applications showcase the superiority of Raman spectroscopy technology in the biomedical field and provide researchers with precise and reliable analytical tools.

In 2010, Raman technology was combined with chemometrics to detect, separate, and identify *Escherichia coli* microbes rapidly. Additionally, more advanced Raman spectroscopy techniques for the visualization analysis of the internal chemical components of microorganisms were applied [54], driving the widespread application of this technology in microbiology. In 2011, Abu-Absi et al. [31] successfully monitored multiple parameters in a bioreactor, including glucose, lactate, ammonium, glutamine, and cell viability. Chen et al. [55] revealed the correlation between *E*. *coli* and surface-enhanced Raman spectroscopy (SERS) shifts, allowing for the quantitative analysis of 13C and 15 N atomic contents in *E*. *coli*. In 2023, Usman et al. [56] utilized SERS combined with machine learning techniques to differentiate Shigella and *E*. *coli* accurately. Recently, Morder et al. [57] utilized SERS to experimentally shorten the offline detection time of lentiviral vector titers to within one day. The real-time monitoring of key indicators such as glucose, protein, and amino acid concentrations in bacterial culture processes has been established by combining Raman technology and chemometrics. Advances in Raman technology, particularly when integrated with chemometrics, have significantly contributed to biological research. A pivotal moment came in 2015 when Kang and colleagues [58] employed high-speed confocal Raman microscopy alongside spherical gold nanoparticles. This innovative approach allowed them to capture rapid morphological changes during cell death at high speed and high resolution, offering new insights into cellular dynamics. Building on this, Wang et al. [59] in 2016 developed a novel method that combined single-cell Raman spectroscopy with stable isotope detection. By analyzing the biochemical fingerprint spectrum of individual microbial cells, they were able to directly interpret the functional roles of these microbes in their natural environments. Furthering the application of Raman spectroscopy in biology, Tao and colleagues [60] used heavy water single-cell Raman spectroscopy in 2017 to scrutinize metabolic activities of oral bacteria at the single-cell level. This technique not only enhanced our understanding of bacterial metabolism but also introduced a new avenue for evaluating antimicrobial effects.

Raman spectroscopy has also been applied to cellular microscopy imaging and cell sorting. In 2018, Chen et al. [61] developed a nano-particle-mediated surface marker expression phenotyping Raman imaging method, combined with microfluidic chip screening of blood samples from xenograft models, to predict the likelihood of cancer metastasis. In 2010, Li et al. [62] detected oral exfoliated cells using near-infrared Raman spectroscopy, demonstrating the ability of Fourier transform Raman spectroscopy (FT-Raman) to distinguish oral squamous cell carcinoma (OSCC), oral leukoplakia (OLK) and normal oral mucosa. Therefore, Raman spectroscopy is particularly effective for cancer diagnostics due to its high specificity and non-invasive nature. The technique provides detailed molecular information about tissue samples without damaging them, allowing for precise identification of cancerous cells and their unique biochemical composition. This level of specificity ensures accurate detection even in heterogeneous tissues. Additionally, the non-invasive nature of Raman spectroscopy means it can be used for real-time analysis during surgical procedures or for minimally invasive biopsies, thereby reducing patient discomfort and recovery times. These advancements in diagnostic capability directly contribute to improved treatment planning by enabling clinicians to tailor therapies to the specific molecular profile of each tumor, ultimately enhancing patient outcomes and survival rates. Their study provides new possibilities for early diagnosis of oral cancer. Esmonde-White et al. [63] comprehensively explored the critical role of Raman spectroscopy technology in biopharmaceuticals. The authors highlighted that the pharmaceutical industry has achieved higher product quality, process intensification, and real-time control with the widespread adoption of quality by design (QbD) and process analytical technology (PAT) frameworks. Unlike some techniques that require physical contact or invasive procedures, Raman spectroscopy can analyze samples through a variety of transparent or semi-transparent materials, such as glass or plastic packaging. This non-invasive nature allows for real-time monitoring without disturbing the sample or process. Goldrick et al. [64] further advanced the application of Raman spectroscopy technology in biopharmaceutical process development. Our research group and other scholars have demonstrated that Raman spectroscopy is a powerful and accurate PAT tool in bioprocess when combined with deep learning technology [65,66].

### 2.3 Application of Raman imaging technology in biomedicine

In drug delivery and nanomedicine, researchers use Raman imaging technology to track the distribution and dynamics of nanoparticles within the body and understand how cells take up and distribute drugs, thereby optimizing drug delivery [67]. With specific nanoparticle labeling, precise imaging and monitoring of pathological tissues such as tumors can be achieved. Raman imaging enables label-free identification and imaging of biomolecules within cells, such as proteins, lipids, and nucleic acids, aiding scientists in understanding the functions and interactions of biomolecules [68]. In conclusion, label-free imaging offers numerous advantages over traditional labeling methods, including non-invasiveness, reduced sample preparation, avoidance of artifacts, real-time capabilities, broader applicability, enhanced sensitivity, and environmental and health benefits. While both approaches have their unique strengths and applications, the growing adoption of label-free techniques highlights their transformative potential in scientific research and clinical diagnostics. For the analysis of organelles and cellular structures, Raman imaging can observe the dynamic changes of organelles and the details of the internal structure of cells, providing essential insights into the study of cell functions and disease mechanisms [69].

In non-invasive diagnostics and disease monitoring, Raman imaging is non-intrusive. Indeed, it can acquire information without destroying the sample, making it suitable for non-invasive diagnostics and real-time monitoring of disease progression [70]. Regarding drug efficacy evaluation and design, by analyzing the distribution and metabolism of drug molecules in biological systems, Raman imaging can assess the therapeutic effects of drugs and provide molecular-level information for drug design [68]. In summary, Raman imaging contributes to drug design by offering molecular-level insights that enhance our understanding of diseases, validate drug targets, monitor therapeutic responses, optimize formulations, personalize treatments, and improve drug delivery system. In biomaterials and tissue engineering, Raman imaging can analyze biomaterials' surface properties and biocompatibility and monitor cell growth and differentiation in tissue engineering [71]. For early disease detection, Raman imaging technology can identify and monitor subtle changes within biological systems, aiding in the early discovery of lesions and diseases, thus enabling early intervention [72]. *In vivo* imaging is also possible with specific Raman probes, allowing for imaging of live biological systems and providing real-time, dynamic information on biological processes [73].

Based on the above discussions, the specific advantages of Raman imaging over alternative techniques such as fluorescence and infrared imaging can be concluded as: Raman imaging can achieve high spatial resolution, often down to the micrometer level, which is particularly advantageous for applications requiring precise localization of chemical components within complex samples; Unlike fluorescence, which relies on broad emission bands and may require multiple labels for different targets, or infrared imaging that primarily detects functional groups rather than specific molecules, Raman imaging can distinguish very similar compounds without the need for extrinsic labels, making it ideal for studying complex biological systems or material compositions. And the non-invasive nature of Raman imaging enables analysis of samples in their native state without the requirement for extensive sample preparation or potential alteration through labeling agents.

Notably, the application of Raman imaging technology in the biomedical field is increasingly widespread, with its non-invasive nature, high specificity, and molecular-level imaging capabilities providing a powerful tool for disease diagnosis, treatment, and biological research. With the continuous advancement of technology, the future integration of Raman imaging with deep learning is expected to play an even more significant role in the biomedical field.

## 3 Artificial intelligence models in Raman spectroscopy and imaging

Raman spectroscopy and imaging are crucial tools that contain a wealth of chemical and material information (e.g., biomolecules, polymers, and nanomaterials). Artificial intelligence, especially deep learning algorithms, can be applied to various applications, such as preprocessing (spectrum denoising), analysis (spectrum analysis, and material classification) and imaging (chemical imaging, and biomedical imaging).

Deep learning models such as CNNs and autoencoders can extract signals from noisy spectral data, thereby improving spectral data quality. Additionally, deep learning models can be trained for qualitative and quantitative analysis of substances, identifying the particular peaks in the spectrum and associating them with chemical substances. Furthermore, deep learning architectures can perform pixel-level analysis on Raman images, achieving segmentation and recognition of regions of interest. At the same time, deep learning models can also learn from Raman spectra or images to classify different types of substances or samples and predict the concentration of unknown samples [74].

### 3.1 CNNs

CNNs are a class of deep neural networks, and widely applied to analyzing visual imagery, which mainly include convolutional layer, pooling layer, and fully connected layer. CNNs have brought about a revolution in the field of artificial intelligence due to their unique architectural design and excellent performance in handling grid-like data. However, large amount of labeled data is required for training and can be a bottleneck in some data-poor areas.

Madsen et al. [75] conducted real-time monitoring for the *in vitro* transaminase-catalyzed synthesis of pharmaceutically relevant amine precursors using Raman spectroscopy and one-dimensional convolutional neural network models. Their approach utilizes Raman spectroscopy technology and model development to monitor biocatalytic reactions. Mozaffari and Tay [76] introduced an anomaly detection scheme for surface-enhanced Raman scattering using a one-dimensional CNNs. The unique model structure can address the data imbalance issues and its application in on-site investigations with portable Raman spectrometers [76,77]. Wang et al. [78] developed a one-dimensional shallow CNNs with an elastic net for quantitative glucose analysis through Raman spectroscopy. The method is characterized by 5% better predictive accuracy and robustness.

**Fig. 2:**
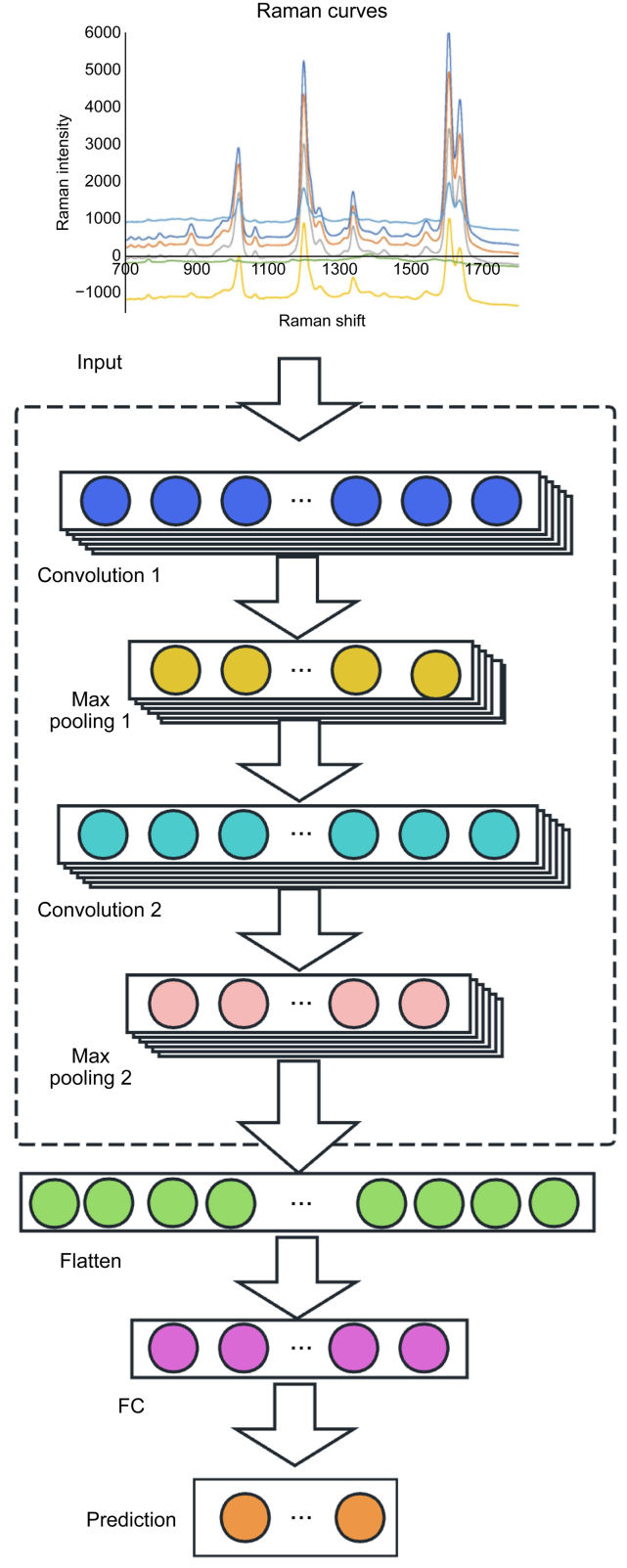
One-dimensional convolutional neural networks (1D-CNNs) architecture. The figure illustrates a simplified 1D-CNN architecture for sequential data processing. It consists of convolutional layers to extract features, followed by max pooling layers to reduce dimensionality. The extracted features are then passed through a fully connected layer to generate predictions. This architecture is efficient for tasks involving sequential data, such as time series analysis and signal processing. Reproduced from Ref. [19] with permission. FC: fully connected layer.

Convolutional layer seeks different scales of feature patterns by performing convolution operations with the input data using convolutional kernels for a given Raman spectroscopy dataset. This helps extract local features and preserve spatial structural information. In a 1D-CNNs, the convolutional kernel slides along one dimension of the input data (usually time series or spectral features) to extract features at different positions [78]. By learning the weight parameters of the convolutional kernels, the convolutional layers can effectively perform feature extraction, thereby capturing frequency shift patterns and peak information within the data. Fig. 2 depicts the structure of a 1D-CNNs, where the output layer of the last one determines the model's function [19].

Pooling layers (max and average pooling), which typically follow convolutional layers, downsample the convolutional output to reduce feature dimensions and computational load while preserving important features [79], which can optimize feature recognition and generalization in Raman spectroscopy applications. Fully connected layers are located at the end of the neural network and map the features extracted from the convolutional and pooling layers to specific category probabilities. Fully connected layers use weight matrices to appropriately combine and transform the feature layers from the previous layers, generating classification or regression results. Activation functions introduce non-linearity to the output of each neuron, such as ReLU, Sigmoid, or Tanh, adding non-linearity to the model and fitting the complexity of the data [80].

Loss functions, commonly the cross-entropy loss function, measure the difference between the model's output and the true labels. Through optimization algorithms like Stochastic Gradient Descent (SGD) or Adam, the loss function is minimized to optimize the model's parameters [81].

### 3.2 LSTM

LSTM networks, first introduced by Hochreiter and Schmidhuber [82] in 1997, are a particular type of recurrent neural network architecture designed to address the issues of vanishing and exploding gradients encountered by traditional recurrent neural networks (RNNs) when processing long sequence data. LSTM networks can effectively capture long-term dependencies in sequence data. However, the design and debugging of the LSTM model can be complex, such as the selection of the appropriate number of layers, the number of units, the setting of the forgetting gate, etc., which requires careful adjustment to achieve the best performance.

Lu et al. [83] proposed a novel method utilizing artificial intelligence and LSTM Raman spectroscopy analysis to rapidly identify bacteria and their resistance genotypes and phenotypes to common antibiotics. The research results demonstrate that their method is highly accurate (89.9%) in bacterial species classification, identification of antibiotic resistance genes, and prediction of resistance phenotypes. Additionally, Wei et al. [84] introduced a multi-scale sequential feature selection method using Raman spectroscopy data and LSTM neural networks for disease classification. Their method was proven faster and more accurate than traditional CNNs since the new method can capture both global and local features. Lastly, Leng et al. [85] combined Raman spectroscopy and FTIR fusion technology with deep learning methods in the form of real-time iterative updates, and proposed a novel cancer prediction method. The research results infer that combining multispectral fusion and deep learning can improve cancer prediction accuracy. Notably, combining deep learning and Raman spectroscopy data has great potential in microbial identification, disease classification, and cancer prediction, offering new research ideas and methods for the medical and biological fields.

**Fig. 3:**
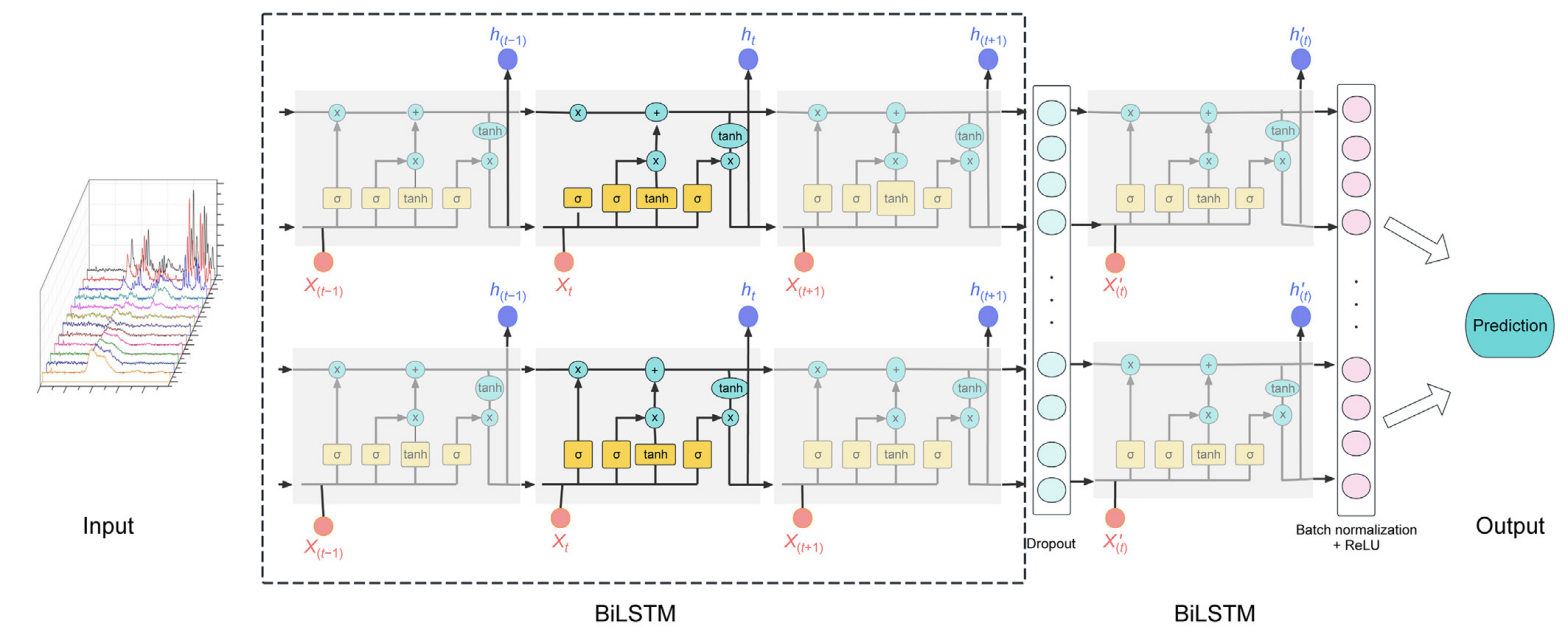
Enhanced bidirectional long short-term memory (EBiLSTM) network architecture. The model processes sequential input data through two BiLSTM layers to capture both past and future context. Dropout is applied to prevent overfitting, and batch normalization with rectified linear unit (ReLU) activation is used to stabilize and enhance learning. The final output is generated for tasks such as sequence prediction or classification. This architecture is effective for modeling complex temporal dependencies in sequential data. σ means that the Sigmoid activation function scales the value to 0–1, the tanh function compresses the value between −1 and 1, and the activation function helps forget gate updates or forget information. ReLU: rectified linear unit; X: input variables; h: hidden layer neurons; tanh: hyperbolic tangent function; σ: sigmoid activation function; t: time.

Bidirectional long short-term memory (EBiLSTM) is a deep learning model that combines the LSTM network structure with the bidirectional information flow, specifically designed for handling time series data [86,87]. The critical components of EBiLSTM are its memory units, which comprise a cell state and a series of gate control units, including the forget, input, and output gates. These gate control units control information inflow, retention, and outflow to capture essential features in sequence data accurately [88]. The EBiLSTM architecture (Fig. 3) comprises forward and backward LSTM components, allowing it to simultaneously consider and learn both forward and backward information of the input data to understand local and global relationships in sequences better. By enhancing the representation and generalization capabilities through bidirectional information flow, EBiLSTM can effectively handle various types of sequence data in monitoring chemical reactions or identifying bacterial resistance. During training, Raman spectroscopy data is input into the EBiLSTM model, which adjusts its parameters to adapt to different classification tasks by learning the patterns and features of the sequence data. EBiLSTM demonstrates robust sequence modeling and feature-capturing capabilities when processing Raman spectroscopy data, effectively understanding the features (e.g., peak shifts, intensity changes) and variations within the spectral data. Specifically, regarding quantitative analysis (e.g., predicting glucose concentrations), EBiLSTM can learn the relationship between concentration and response in the spectrum, thereby predicting and estimating the specific compound concentrations in samples. Considering qualitative analysis (e.g., identifying bacterial strains), EBiLSTM can identify and classify differences between various samples, rapidly identifying and classifying individual samples or substances.

### 3.3 GANs

In GANs, the generator produces realistic data, while the discriminator aims to distinguish the generated and real data, and has been widely applied to Raman spectroscopy (e.g., anomaly detection or data augmentation). The generator learns how to distribute real data to create synthetic data similar to real data, while the discriminator classifies the data based on the difference between the data generated by the generator and real data. The generator and discriminator continuously compete and improve each other's performance to create realistic data. However, it is difficult to judge when the training stops and whether it has reached an optimal state because the loss function values of the discriminator and generator affect each other and may fluctuate, making convergence difficult to determine.

For the common problem of low accuracy of the model, Wang et al. [89] introduced a Raman spectroscopy model transfer method based on Cycle-GAN to address the spectral data mismatch between different instruments. This approach achieved domain transfer of spectral data with a cosine similarity exceeding 99%, which means the model can be used in different domains. Besides, Hu et al. [90] introduced a GAN super-resolution algorithm to enhance Raman interferometry images and improve signal-to-noise ratio. This method stores low-resolution interferometry images in high-resolution images, enabling miniaturization and portability for point-to-point testing in Raman spectroscopy users. Zheng et al. [91] combined near-infrared spectroscopy technology with bidirectional GAN to identify multi-class of drugs. This technique improves the performance of the regression model through data augmentation, addressing the limited training data issue.

**Fig. 4:**
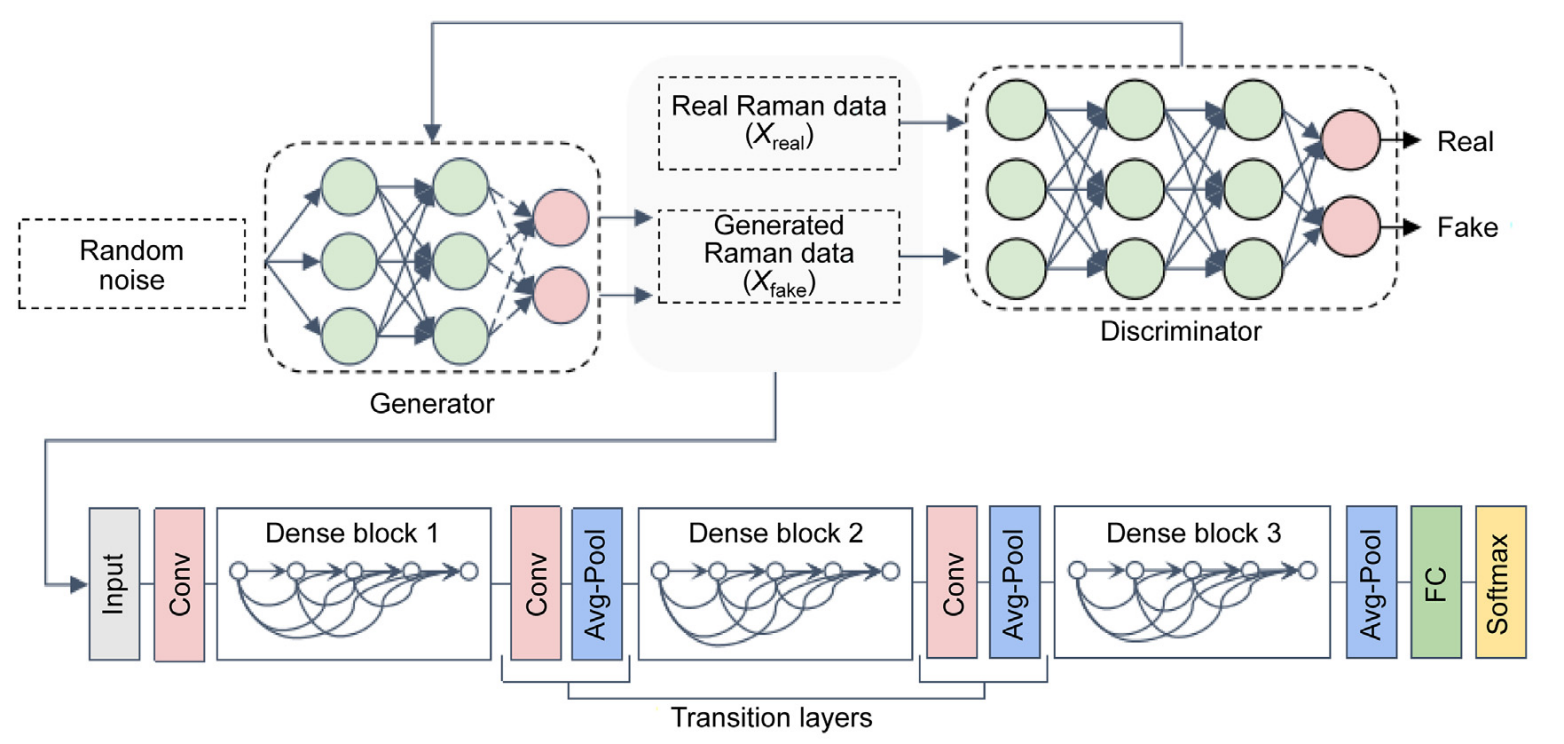
Generative adversarial network (GAN)-convolutional neural network (CNN) model framework. It integrates a GAN with a CNN. The generator creates synthetic Raman data (*X*_fake_) from random noise, while the discriminator distinguishes between real (*X*_real_) and generated data. The CNN component includes dense blocks, convolutional layers, average pooling, and transition layers for feature extraction and dimensionality reduction. The final fully connected layer with softmax activation produces the output. This framework is designed for advanced data generation and classification tasks. Conv: convolutional layers; Avg-Pool: average pooling; FC: fully connected layer.

Generating many ‘synthetic spectra’ by GAN reduces the data volume requirement for training classification tasks through large data sets practiced by CNN (Fig. 4). The generated synthetic data expands the training set, increases data diversity, and improves the model's generalization ability and performance. Extraction and classification GAN models can learn high-level feature representations, which are beneficial for feature extraction and classification of spectroscopy data, thus distinguishing overlapping spectral peaks or identifying subtle spectral shifts. The generator can extract essential features from the spectroscopy data for subsequent classification tasks. Furthermore, regularities and implicit information within the data can be discovered through GAN models, providing valuable features for subsequent analysis [92]. A specific example of a “valuable feature” identified by a GAN model in Raman spectroscopy could be the detection of subtle spectral shifts associated with different phases of a chemical reaction. And a GAN model was trained on a large dataset of Raman spectra collected at various stages of the reaction. The model learned to recognize and emphasize the spectral features that correlate with the formation of intermediate species and the transition between different reaction phases, leading to enhanced analysis and prediction accuracy.

### 3.4 GNNs

Graph isomorphism networks originated in the 1970s and have been proposed by several researchers. However, the modern graph isomorphism network models, particularly the perceptron model proposed by Minsky in 1975, laid the foundation for developing GNNs [93]. The core principle of the GNN lies in effectively learning representations of graph-structured data, which comprise a set of nodes (vertices) and edges (or links) that connect these nodes [94]. However, the training process of GNNs usually involves complex matrix operation and message passing mechanism, which requires a large amount of computation. With the increase of the scale of the graph, the calculation time will increase significantly, resulting in low training efficiency, which is difficult to meet the requirements of real time in practical applications.

**Fig. 5:**
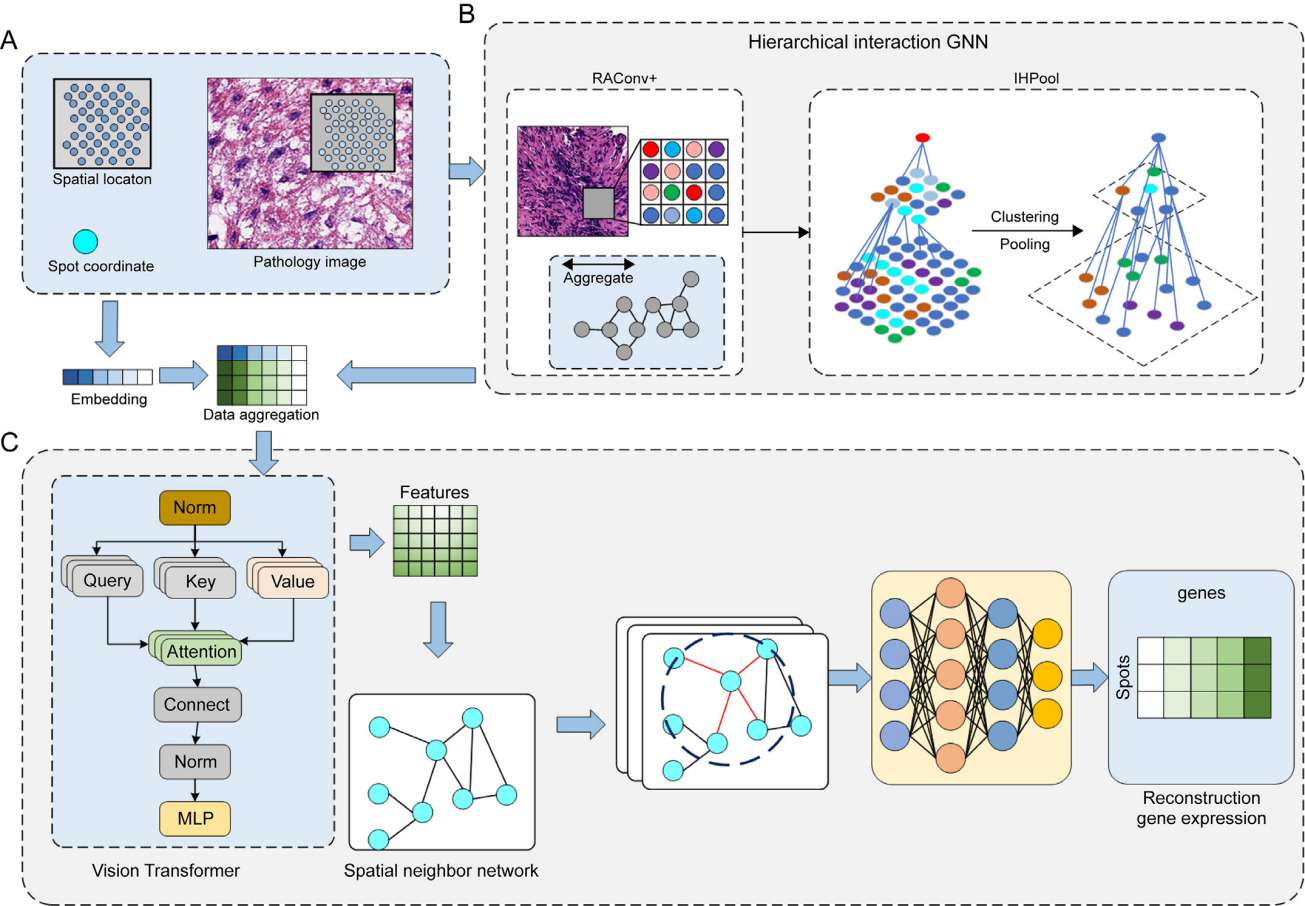
Cellular structure detection algorithm based on graph neural networks (GNN) and self-attention mechanism. The figure illustrates a cellular structure detection algorithm that integrates GNN and self-attention mechanisms. The process begins with spatial location and clustering of cellular data, followed by pooling to aggregate features. The algorithm employs RAConv+ for advanced feature extraction and reconstruction. A Vision Transformer is utilized to enhance detection accuracy through self-attention, capturing intricate cellular patterns. This approach enables precise identification and analysis of cellular structures in complex biological datasets.

GNNs encompass several components: node feature representation, graph convolution, message passing mechanisms, multi-layer perceptron (MLP), and pooling and graph readout operations [95]. The core mechanism of GNNs is message passing, where each node aggregates information from its neighboring nodes. This process typically includes two primary operations. The first is information collection, where nodes gather information from their neighbors [96]. This can be achieved in various ways, such as simple feature concatenation, weighted summation, or more complex transformations. The other is node update, where nodes update their feature representations based on the collected information. This usually involves a learnable transformation, such as one or more fully connected layers (also known as neural network layers) [97]. Fig. 5 reveals that the GNN network identifies the structural features between cells of pathological tissues and uses the self-attention mechanism to understand the features of the topological structure graph and predict the expression of recombinant genes.

The characteristics of GNNs in Raman imaging can assist numerous applications in the biomedical field, such as analysis of molecular interaction networks and disease diagnosis, and how GNNs improve the accuracy of tissue classification or enhance the understanding of drug-target interactions can be found in Refs. [98,99]. Raman imaging generates vast spectral data for data interpretation and feature extraction, with each data point containing rich chemical information. GNNs can treat these data points as graph nodes and establish edges based on spectral similarity. Through training, GNNs can learn the relationships between nodes, thus extracting features that help distinguish different biomolecules. For instance, in studies considering drug-protein interactions, GNNs extract features of drug molecules binding to proteins from Raman spectra, thereby predicting the drug's binding affinity and mechanism of action [100].

GNNs can construct graphs of molecular interaction networks, where nodes represent different molecules and edges denote interactions between them. By analyzing Raman imaging data, GNNs can identify intermolecular patterns and strengths. For instance, when studying cellular signaling pathways, GNNs can help reveal which molecules are key signaling nodes and how their interactions influence cellular behavior [101]. Different biological tissue structures in Raman imaging give rise to distinct spectral features. GNNs can learn and utilize these features to identify and classify tissue structures [102]. For example, in the early skin cancer diagnosis, GNNs can differentiate between normal and cancerous tissue by analyzing Raman imaging data. GNNs can identify the early stages of carcinogenesis [103]. Besides, GNNs can integrate Raman imaging data with other clinical information, such as a patient's medical history and laboratory test results, to enhance the accuracy of disease diagnosis. Hence, in cancer diagnosis, GNNs can analyze Raman imaging data from tumor tissues. When combined with a patient's genetic information, it can predict the malignant behavior of the tumor and provide the patient's prognosis [104].

In drug delivery research, GNNs can leverage Raman imaging data to monitor the distribution of drug nanoparticles within the body and drug release. By constructing a graph model of the drug delivery pathway, GNNs can predict the accumulation of the drug in specific tissues or cells, thereby assessing its therapeutic effects [105].

### 3.5 Transformer model

The Transformer is a deep learning model based on the self-attention mechanism, primarily used for processing sequential data, such as natural language processing (NLP) and biomedical data analysis tasks. It was proposed by Vaswani et al. [106] in 2017 and has achieved significant performance improvements on several tasks. The core principle of the Transformer is based on the structure of encoders (e.g., processes spectral data) and decoders (generates predictions or classifications), which are its two main components [107]. The encoder transforms the input sequence into a continuous representation, while the decoder generates the target sequence based on the encoder's output and previous outputs [108]. However, the internal structure and working mechanism of the Transformer model are relatively complex, involving multiple components such as multi-head attention and residual connection, which makes it difficult to interpret the output result of the model intuitively and make users difficult to understand how the model makes decisions based on the input data.

The self-attention mechanism is the core of the Transformer model, allowing it to consider all positions within a sequence simultaneously while capturing long-range dependencies within the sequence [109]. This mechanism calculates the weights between each element and every other element in the sequence to determine their degree of association. The Transformer model employs a multi-head attention mechanism to capture different types of relationships by dividing the self-attention mechanism into multiple “heads”, each learning to capture other relationships [110]. The outputs of all heads are then concatenated to form the final attention representation. For example, the self-attention mechanism allows the model to focus on different parts of the input sequence and compute their interactions. This helps in capturing global dependencies and subtle variations in the spectral data, multi-head attention further improves the analysis by allowing the model to jointly attend to information from different representation subspaces at different positions. This enhances the ability to distinguish subtle variations in overlapping peaks. Since the Transformer model cannot capture positional information within the sequence, positional encoding is introduced. Positional encoding adds sinusoidal functions of different frequencies to the input data, which helps the model to learn the positional information of each frequency point in the sequence. After the self-attention layer, the Transformer uses a feedforward network to perform non-linear transformations on each element, allowing the model to learn more complex representations. It should be noted that the Transformer model introduces residual connections between each sub-layer (self-attention and feedforward network) to address the vanishing gradient problem in training deep networks [111]. Additionally, the model uses layer normalization, Layer norm is applied after each fully connected layer to normalize the activations. This helps in stabilizing the training process and improving the overall performance of the model on Raman spectral data. A significant advantage of the Transformer model over RNN is its ability to process all elements in a sequence in parallel, substantially increasing training speed. Through techniques such as the self-attention mechanism, multi-head attention, and positional encoding, the Transformer model effectively processes sequential data and captures long-range dependencies, achieving significant performance improvements on tasks involving sequence data processing [112].

**Fig. 6:**
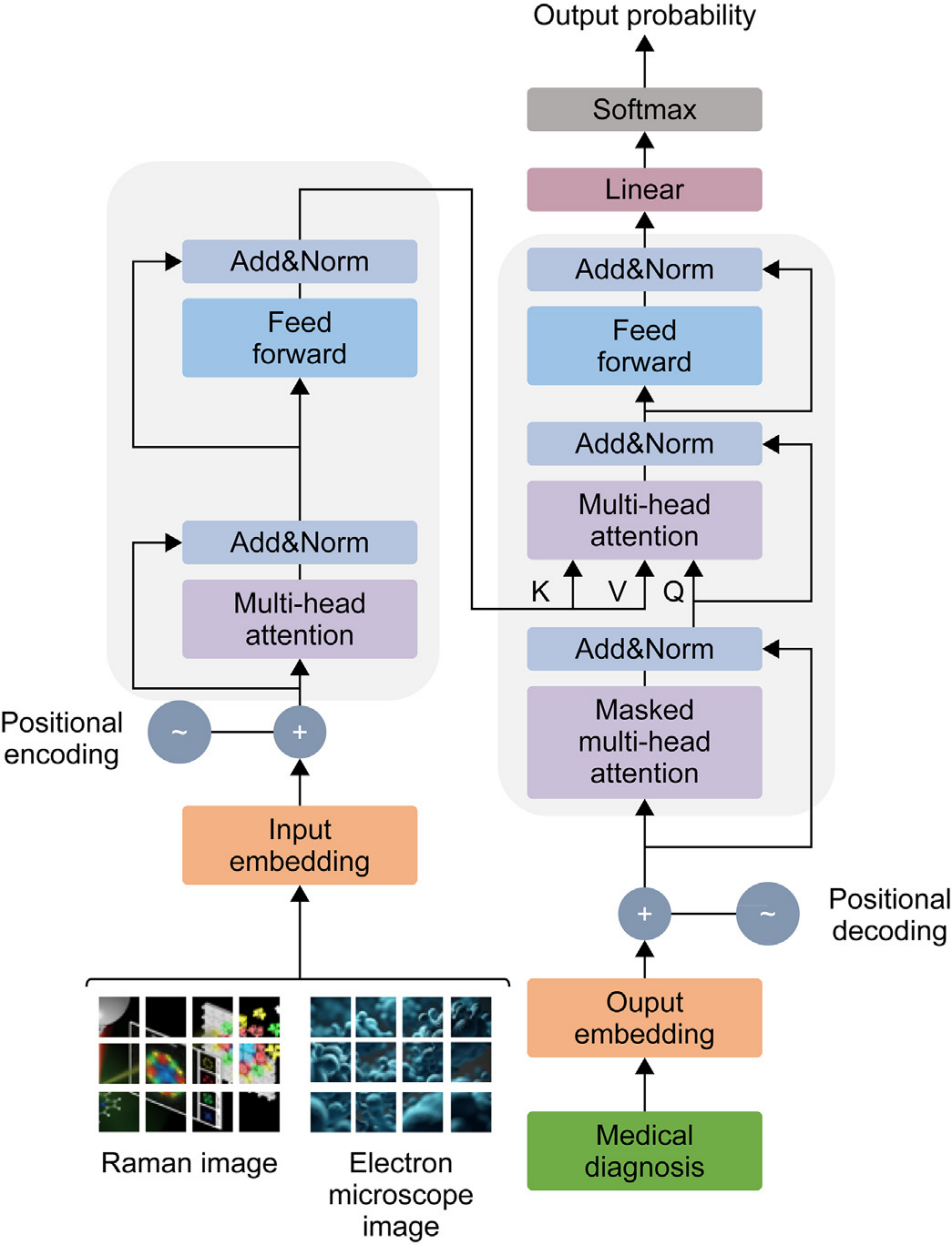
Transformer-based multi-modal feature fusion for aided diagnosis. The figure presents a Transformer-based architecture designed for multi-modal feature fusion in medical diagnosis. The model begins with input and positional embeddings, followed by masked multi-head attention and standard multi-head attention layers to capture contextual relationships. Add&normalization layers stabilize training, while feed-forward networks process the features. The output embedding is passed through a linear layer and softmax activation to generate diagnostic probabilities. This framework enhances the integration of diverse data modalities for improved medical decision-making. Reproduced from Ref. [106] with permission. K: key; V: value; Q: query.

The Transformer model has also been increasingly applied to computer vision and biomedical data analysis, including Raman imaging in the biomedical field [113]. As depicted in Fig. 6, the multi-head attention mechanism of the Transformer model is used to fuse multi-modal molecular structure images, and the data information of multiple devices is integrated to realize the purpose of medical detection and auxiliary diagnosis [106]. Precisely, the self-attention mechanism can capture relationships between different parts of the image, thereby improving classification accuracy, e.g., in cancer diagnosis, the Transformer can distinguish benign and malignant tumors. Raman imaging data typically contains complex chemical and spectral information regarding feature extraction and representation learning. The Transformer model can learn high-level representations of these data, extracting deep features useful for subsequent analysis [114]. For multi-modal data fusion in biomedical research, combining data from different sources, such as Raman imaging, fluorescence imaging, electron microscopy, and others, is often necessary. In this case, the Transformer model can effectively process and integrate multi-modal data, providing a more comprehensive analysis of biological tissues [115]. A specific example of multi-modal data fusion involving Raman imaging and other modalities is the combination of Raman spectroscopy with fluorescence microscopy in cancer tissue analysis. This fusion leverages the complementary strengths of both techniques to provide a more comprehensive understanding of the biochemical and morphological characteristics of tissues.

In the advanced research field of drug discovery and molecular design, Transformer models are utilized to perform deep analysis on the Raman spectral data of drug molecules, enabling precise predictions of the binding affinities between drug molecules and target proteins. Through its advanced self-attention mechanism, the Transformer can effectively extract high-dimensional features of drug molecules, providing a powerful predictive tool for drug screening and facilitating the efficient identification of drug candidate molecules with potential activity [116]. Expanding into biological tissue analysis and disease diagnosis, Transformer model can parse complex patterns within Raman imaging data, accurately identifying and classifying different cell types and pathological states within biological tissues [117]. For example, by leveraging the self-attention mechanism, Transformers can effectively weigh the importance of different spectral features, enhancing the model's ability to distinguish between healthy and diseased tissues.

At the cell activity monitoring level, the Transformer has been employed to analyze the Raman spectral sequences of intracellular molecules, enabling real-time monitoring of dynamic changes in intracellular molecules. Precisely, the model can capture changes in intracellular enzyme activity, fluctuations in metabolic pathways, and other vital physiological parameters, providing valuable information for studying cellular physiology and pathological processes [118]. Regarding image reconstruction and enhancement, applying the Transformer model further expands the scope of application for Raman imaging technology [119].

## 4 Prospects of deep learning guided Raman spectroscopy

The application prospects of deep learning technologies in Raman spectroscopy analysis are vast [120,121], with semi-supervised learning, reinforcement learning, transfer learning, and Transformer models being particularly significant. Applying these technologies will drive the development of Raman spectroscopy and imaging in the biopharmaceutical field, enhancing the accuracy and efficiency of analysis.

Semi-supervised learning effectively utilizes unlabeled data to aid in training labeled data, such as identifying chemical compositions or detecting impurities, improving models' generalization ability and robustness. In Raman spectroscopy analysis, semi-supervised learning combined with a small amount of labeled data can enhance models' performance and predictive capabilities. Future research will further explore the application of semi-supervised learning algorithms in Raman spectroscopy analysis to fully utilize the potential of unlabeled data (e.g., noisy data or inconsistent labeling) [122].

Reinforcement learning learns the optimal strategy through trial and error. It can be used in Raman spectroscopy analysis to automatically optimize spectral preprocessing steps, such as feature selection and parameter tuning. Through reinforcement learning, more efficient spectral data processing and analysis can be achieved, enhancing the performance and accuracy of models. Future research will develop reinforcement learning algorithms specifically for Raman spectroscopy analysis to improve model prediction and robustness, such as computational cost or the need for well-defined reward functions.

Transfer learning can utilize knowledge learned in the source domain to improve performance in the target domain. In Raman spectroscopy analysis, transfer learning can quickly adapt pre-trained deep learning models to new spectral data, such as adapting a model trained on pharmaceutical data to analyze biological tissues. The models' generalization ability and robustness can be improved through transfer learning, accelerating the training speed of new tasks (e.g., classification, and regression). Future research will explore transfer learning strategies in Raman spectroscopy analysis to enhance model performance and predictive capabilities [123].

Transformer models perform excellently in processing sequential data and can effectively extract and analyze critical features in Raman spectroscopy data. Additionally, Transformer models can be used for tasks such as image segmentation and object recognition, improving the accuracy and robustness of Raman spectroscopy and imaging analysis. Future research will further explore the application of Transformer models in Raman spectroscopy analysis to achieve more efficient and accurate analysis results.

In summary, the application prospects of deep learning technologies in Raman spectroscopy analysis are very promising. Semi-supervised learning, reinforcement learning, transfer learning, and Transformer models will bring new development opportunities to Raman spectroscopy analysis. As these technologies continue to develop and optimize, their applications in Raman spectroscopy analysis will become more widespread and profound, bringing more innovation and breakthroughs to research and applications in the biopharmaceutical field.

## 5 Conclusions

Raman spectroscopy has become a crucial analytical tool in drug development and pharmaceutical processes. This technology, known for its high sensitivity and resolution in drug molecular structure analysis and component identification, accelerates drug development and enhances quality control efficiency. In biomedical research and clinical diagnostics, Raman spectroscopy provides a new perspective and method for disease early diagnosis and treatment through high-resolution component mapping. Its application in drug metabolism research, especially in studying drug-biomolecule interactions, is important for optimizing drug design and improving treatment effectiveness. Additionally, the real-time monitoring capability of Raman spectroscopy in pharmaceutical processes ensures process efficiency, safety, and the consistency of the final product quality.

The application of deep learning technologies in chemometrics has revolutionized traditional analytical chemistry. By leveraging deep learning models such as CNNs, LSTM, GANs, GNNs, and Transformer models, chemometrics has significantly improved data processing, feature extraction, and model optimization. Deep learning models can handle large-scale and high-dimensional datasets, reducing the workload of manual feature engineering. By automatically learning complex patterns and features within the data, deep learning models enhance the efficiency of chemometric analysis. Regarding feature extraction, deep learning models, especially CNNs, excel in extracting features directly from raw data, such as spectral data. These features better represent critical information in chemometrics, thereby improving prediction accuracy and robustness of models.

By adjusting network structures and parameters, deep learning models optimize the performance of chemometric models. These models can quickly adapt to new datasets and tasks through transfer learning and fine-tuning strategies. Additionally, they excel in predictive analytics, which applies to quantitative analysis and qualitative identification. These models have broad application potential in biopharmaceuticals, materials science, and environmental monitoring. Furthermore, deep learning models have an advantage in image processing and can be used for chemical imaging and microscope image analysis, providing a new perspective and tools for chemometrics. Although deep learning models perform well in prediction, they are often considered “black boxes”. Hence, to enhance the interpretability of models, researchers are exploring interpretable deep learning methods such as attention mechanisms and ensemble learning.

## CRediT authorship contribution statement

**Yuan Liu:** Writing – original draft, Methodology, Investigation, Formal analysis. **Sitong Chen:** Writing – original draft, Visualization, Data curation. **Xiaomin Xiong:** Writing – original draft, Investigation, Data curation. **Zhenguo Wen:** Writing – review & editing, Writing – original draft, Supervision, Project administration, Investigation, Funding acquisition, Conceptualization. **Long Zhao:** Data curation, Formal analysis, Software. **Bo Xu:** Writing – review & editing, Supervision. **Qianjin Guo:** Writing – review & editing, Supervision. **Jianye Xia:** Writing – review & editing, Supervision. **Jianfeng Pei:** Investigation, Supervision, Writing – review & editing.

## Declaration of competing interest

The authors declare that there are no conflicts of interest.
